# Diabetes Mellitus as a Risk Factor in Recurrent Vestibular Disorders: An Update

**DOI:** 10.3390/audiolres16040115

**Published:** 2026-08-06

**Authors:** Athena Eliana Arsie, Carlotta Muneretto, Matteo Seno, Marta Gaffeo, Luca Sacchetto, Daniele Monzani, Silvia Palma

**Affiliations:** 1 Otolaryngology-Head and Neck Surgery Department, University of Verona, 37129 Verona, Italy; athenaeliana.arsie@univr.it (A.E.A.); matteo.seno@studenti.univr.it (M.S.); marta.gaffeo@studenti.univr.it (M.G.); luca.sacchetto@univr.it (L.S.); daniele.monzani@univr.it (D.M.); 2 Audiology, Primary Care Department, AUSL Modena, 41121 Modena, Italy

**Keywords:** benign paroxysmal positional vertigo, diabetes mellitus, vestibular disorders, vertigo

## Abstract

**Background/Objectives**: Diabetes mellitus (DM) has been proposed as a potential risk factor for vestibular dysfunction, although the evidence remains heterogeneous. This review was prompted by the increasing recognition in clinical practice of a frequent coexistence between DM and recurrent vestibular disorders. It aims to examine the most recent available literature investigating the association between DM and the most common causes of recurrent vestibular disorders, thereby providing clinicians with additional insights for early diagnosis and risk stratification. **Methods**: A systematic review was conducted according to PRISMA guidelines with SWiM-based narrative synthesis. PubMed, Scopus, and Google Scholar were searched for studies (2016–2026) including adult diabetic patients with vestibular disorders. Risk of bias was assessed using NOS, RoB2, and JBI tools. **Results**: Twenty-nine studies met the inclusion criteria, but only nineteen were focused explicitly on recurrent vestibular disease. Diabetes was consistently associated with higher prevalence and recurrence of benign paroxysmal positional vertigo (BPPV), more persistent vestibular symptoms, and worse balance outcomes. Associations with other recurrent vestibular disorders was scarce and high heterogeneity limited quantitative synthesis. **Conclusions**: The available literature suggests that DM increases the vulnerability of the vestibular system in BPPV cases while data regarding other vestibular disorders, including VM and MD, are still limited. More prospective studies investigating the link between diabetes and recurrent vestibular disorders are needed, particularly through specific indicators of evolutionary picture of diabetes.

## 1. Introduction

Diabetes mellitus (DM) is one of the most prevalent chronic metabolic disorders worldwide, with a rapidly increasing global prevalence, and it is associated with a wide range of microvascular, macrovascular, and neurological complications [1,2,3]. DM is characterized by persistent hyperglycemia and impaired carbohydrate, protein, and lipid metabolism resulting from insulin dysregulation. Beyond its well-established effects on the retina, kidneys, and peripheral nervous system, increasing evidence suggests that DM may also impair vestibular function, contributing to dizziness, imbalance, and an increased risk of falls, particularly among older adults. Although the underlying mechanisms have not yet been fully elucidated, current evidence suggests that vestibular apparatus may be affected at multiple anatomical levels [4], involving both the peripheral and central vestibular systems. Since the vestibular organs are supplied by the labyrinthine artery, which is a terminal vessel lacking significant collateral circulation, vestibular sensory structures are particularly susceptible to ischemic damage resulting from diabetes-related endothelial dysfunction [5,6]. Chronic hyperglycemia promotes oxidative stress and metabolic dysregulation, leading to degeneration of vestibular hair cells and structural alterations of the myelin sheath of the vestibulocochlear nerve [2,7], while elevated glucose concentrations may also alter the biochemical composition of the endolymphatic compartment, thereby promoting otolith detachment and facilitating the development of vestibular impairment [6,8,9]. Vestibular disorders are a frequent reason for medical consultation for both general practitioners and ENT specialists. Among peripheral vestibular disorders, benign paroxysmal positional vertigo (BPPV), vestibular migraine (VM), and Ménière’s disease (MD) are the most common causes of recurrent vertigo [2,8,10]. As the association between DM and the peripheral vestibular system has been investigated for many years, epidemiological studies indicate that individuals with diabetes have a significantly higher likelihood—up to 70% greater—of developing vestibular dysfunction than non-diabetic individuals [1,11,12,13,14,15]. In particular, diabetes has been identified as an important risk factor for BPPV recurrence [8,9,16] and as a negative prognostic factor influencing vertigo outcomes in patients with VM [2]. Therefore, DM may act as both a predisposing and an aggravating factor in vestibular disorders [6,16]. Significantly, recurrences not only increase the risk of falls, especially among older adults, but also exacerbate psychological conditions such as anxiety and depression, thereby reducing patients’ well-being. For these reasons, early identification of risk factors is important to enable timely intervention [9]. This review was prompted by the increasing recognition in clinical practice of a frequent coexistence between diabetes mellitus (DM) and recurrent vestibular disorders. It aims to examine the most recent available literature investigating the association between DM and the most common causes of recurrent vestibular disorders, thereby providing clinicians with additional insights for early diagnosis and risk stratification.

## 2. Materials and Methods

This systematic review was conducted and reported in accordance with the PRISMA 2020 Statement. The completed PRISMA checklist is provided as Supplementary Material (Supplementary File S1). Due to the absence of a meta-analysis, the synthesis of the findings was reported following the SWiM recommendations [17]. A literature search was conducted across the major scientific databases, including PubMed, Scopus, and Google Scholar, using predefined search strings that combined terms related to vestibular disorders—including VM, BPPV, MD—with terms related to diabetes mellitus. The complete search strategies are provided in Appendix A. Filters were applied to exclude studies involving presbycusis, animal models, and in vitro experiments. Only studies published between January 2016 and April 2026 were considered. The search was restricted to this period to provide an up-to-date overview of the current evidence regarding the association between DM and vestibular disorders.

Inclusion criteria were as follows: studies published between 2016 and 2026; studies involving adult participants diagnosed with DM; studies involving patients with BPPV, VM, and MD. Prospective, retrospective, observational, and cross-sectional studies, as well as randomized controlled trials, were included.

Exclusion criteria were in vitro animal studies; case reports; meta-analyses; and review articles.

**Figure 1:**
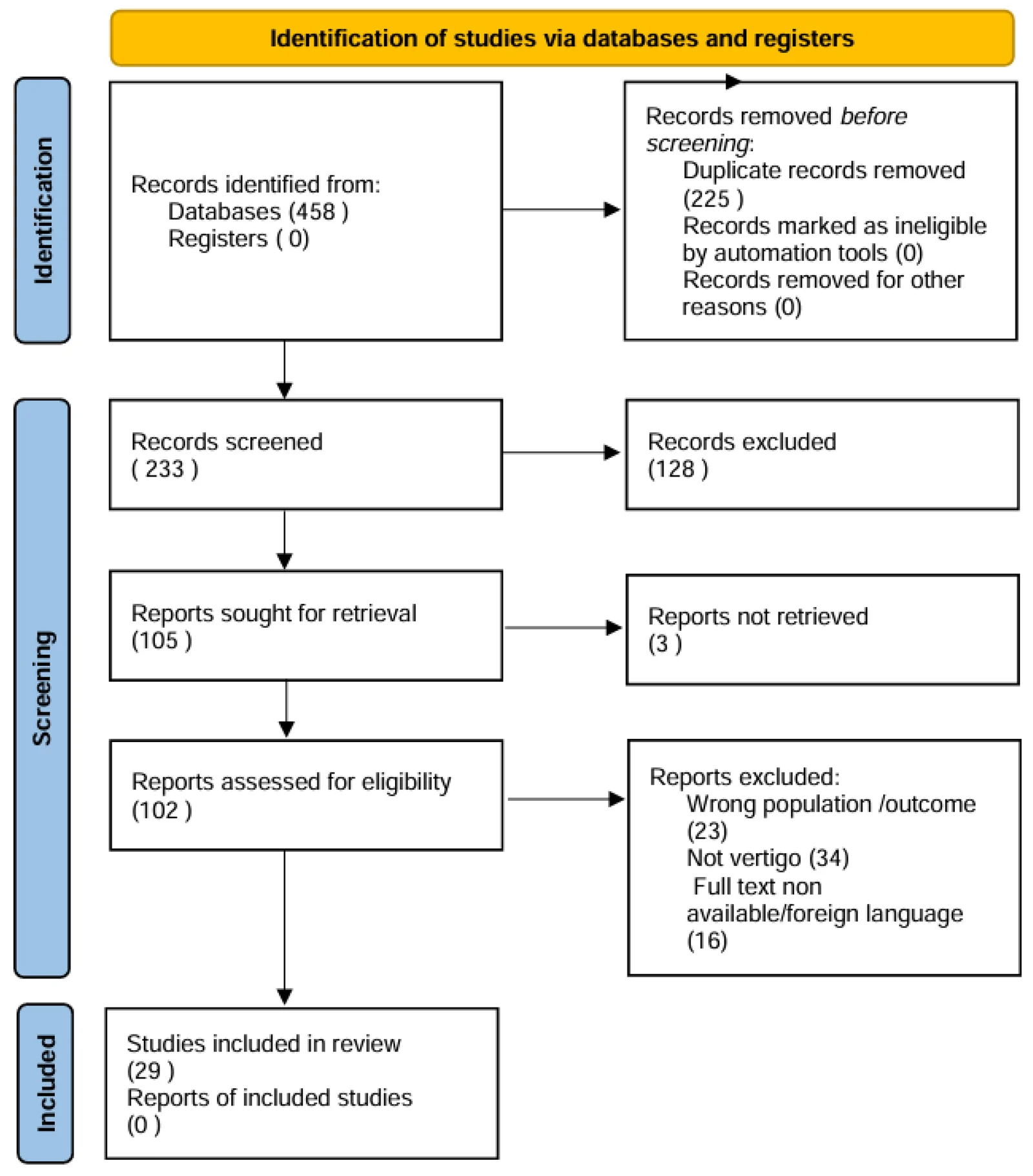
Study selection process.

Article screening and full-text assessment were independently performed by five reviewers in a blinded manner. Any disagreements were resolved through discussion and consensus. The entire selection process was conducted systematically under the supervision of a senior reviewer and with the support of the Rayyan software platform [18]. A structured data extraction process was carried out using a shared database. Following the article screening and full-text assessment, the extracted variables included the author, year of publication, and country of origin. The numbers of patients with diabetes and those presenting with vertigo were also recorded. Outcomes were analyzed descriptively. The collected information focused on the prevalence of recurrent vestibular disorders, the risk of recurrence, and audiovestibular outcomes in patients with diabetes. Risk of bias was assessed according to study design using validated appraisal tools. The Newcastle–Ottawa Scale (NOS) was applied to retrospective, prospective, and case–control studies; the Cochrane Risk of Bias 2 (RoB 2) tool was used for randomized controlled trials; and the Joanna Briggs Institute (JBI) Critical Appraisal Checklist was used for cross-sectional studies. The selection process is summarized in Figure 1. The literature search identified 458 records. After duplicate removal, 233 studies were screened. Of these, 200 were excluded based on the predefined inclusion and exclusion criteria, and the full texts of three studies could not be retrieved. Following the systematic screening process, 29 studies were included in the final review.

## 3. Results

Nineteen of the studies that met the inclusion criteria specifically explored the link between diabetes and vestibular disorders [1,2,3,6,7,8,9,15,16,19,20,21,22,23,24,25,26,27,28]. Overall, the total number of diabetic patients included was 188.682; of those, 169.640 were affected by vestibular disorders while 5544 were explicitly indicated as affected by both conditions as, in a few cases, only percentages were reported and diabetes and vestibular disorders were described separately without specifying the exact number of patients affected by both conditions.

The type of DM, the kind of anti-diabetic treatment, or the presence of diabetes-related complications were not included in the descriptive synthesis, as not all the studies provided these details.

Ten studies [5,29,30,31,32,33,34,35,36,37] were identified as being mainly related to sudden sensorineural hearing loss (SSNHL) and, since they met the predefined selection criteria, were included and analyzed in a distinct paragraph of this section. Globally, the number of subjects included in these studies was 14778.

Overall, 20 studies were retrospective studies, 4 were case–control studies, 2 were prospective studies, 2 were randomized controlled trials, and 1 was a cross-sectional study. Substantial clinical and methodological heterogeneity across study designs, populations, exposures, and outcome definitions precluded meaningful quantitative pooling and therefore did not allow a meta-analysis.

Risk of bias was assessed according to study design using validated appraisal tools.

The Newcastle–Ottawa Scale (NOS) was applied to retrospective cohort, case–control, and database-based studies, evaluating the selection process, the comparability and the outcomes and exposure (see also Table 1). National database studies [1] achieved the highest scores for selection and comparability due to their large sample sizes and the use of propensity score matching while single-center studies lost points because of the absence of external control groups and the relatively high proportion of patients lost to follow-up. Most observational studies achieved high or very high NOS scores, with several studies scoring 7/9 or higher.

RoB 2 Assessment. Both studies employed appropriate randomization procedures, including lottery-based or stratified randomization methods (see also Table 2). However, in Shaphe et al. [15], although outcome assessors were blinded, neither the physiotherapist performing the maneuver nor the participants could be blinded due to the nature of vestibular rehabilitation interventions. For this reason, the domain “deviations from intended interventions” was judged as presenting some concerns regarding performance bias.

In the Johanna Briggs Institute (JBI) Assessment (see also Table 3), Bazoni et al. [22] and D’Silva et al. [7] demonstrated strong methodological quality, clearly defining diagnostic criteria (e.g., Dix–Hallpike test) and employing objective exposure measurements such as biochemical assays, accelerometry, HbA1c levels, and neuropathy assessments. In contrast, Sreenivas et al., described as an analytical descriptive study, did not clearly identify a control group and lacked strategies to address potential clinical confounders, reducing the overall robustness of its conclusions [20].

Concerning Liu’s study [6], according to STROBE-MR principles [38], this study demonstrated a low risk of bias due to the use of large independent cohorts and multiple sensitivity analyses to assess pleiotropy.

Regarding Hui’s study [16], the primary source of bias relates to the quality of the original public datasets (GEO database) [17]. Nevertheless, the analytical framework was strengthened by experimental validation using quantitative PCR (qPCR), supporting the reliability of the findings.

Detailed characteristics of the 19 studies exploring, specifically, the link between diabetes and recurrent vestibular disorders are presented in Table 4.

Most of the studies addressed the relationship between DM and BPPV, whereas data on other vestibular disorders were scarce. Only in three studies was DM not recognized as a risk factor [21,22,34]. No sex-based predominance was observed, except in two studies [2,23]. Overall, it is suggested that DM acts as a critical predictor of persistence and severity of vertiginous symptoms, negatively influencing prognosis in both BPPV and VM.

In a few studies of the same author [7,27,28], patients with both diabetes and BPPV showed significantly higher body sway values compared with controls and diabetic-only subjects, as well as a significantly higher frequency of abnormal cVEMP responses (suggesting saccular dysfunction) in diabetic, BPPV, and combined BPPV-DM groups compared with healthy controls. An increased risk of falls was also reported. DM was identified as a predictor of recurrence of vestibular symptoms in three studies [8,9,20].

Several studies incorporated DM into predictive nomograms for patients with BPPV [6,8,26]. Tang, for example, developed and validated a nomogram to estimate the risk of BPPV recurrence in middle-aged and older adults, identifying diabetes as one of the key predictors together with age, serum uric acid, homocysteine, migraine, anxiety, and insomnia [8].

Concerning the studies that investigated the influence of diabetes on inner ear involvement, SSNHL has been regarded as a clinical manifestation sharing pathophysiological mechanisms with vestibular impairment. The association with vertigo has been indicated as a crucial clinical indicator, reflecting the involvement of both the cochlear and vestibular components of the inner ear [33,34]. In this regard, vertigo was observed more frequently in patients with profound hearing loss than in those with moderate hearing loss (42.86% vs. 17.31%) [32]. Elderly were more likely to present both hearing and vestibular impairment [34].

## 4. Discussion

Overall, the analysis of the more recent literature suggests an association between DM and BPPV while articles about other frequent recurrent vestibular disorders such as VM and MD, are scarce. The prevalence of BPPV is very common in older adults, even though symptoms are less specific in respect of younger populations and probably this reason justifies the discrepancy.

Most studies converge on the finding that diabetic patients have a higher prevalence of BPPV, increased recurrence rates, more persistent symptoms, and overall poorer postural balance outcomes with residual dizziness. Major evidence in this regard comes from a study that analyzed a large Taiwanese cohort [1], but it has also been confirmed by other experiences [7,20,25,27,28]. This pattern is highly consistent, and diabetes does not appear to merely coexist with BPPV, rather to actively contribute to its instability and recurrence of symptoms. This convergence emerges, even though retrospective cohort, case–control, and database-based studies have an higher risk of bias compared to randomized or prospective studies.

Various possible explanations have been proposed, as a result of more factors such as hyperglycemia, increase in reactive oxygen species and reduced antioxidant protective system. Microvascular damage may lead to cochlear and labyrinthine ischemia, with reduced vestibular perfusion and the consequent impairment of the otolithic organs [39]. Cochlear microcirculatory alterations result in changes in the ultrastructure of the inner ear capillaries such as thickening of the basilar membrane and atrophy of the stria vascularis. Neuropathy leads to impaired vestibulo-proprioceptive integration, reduced postural control, and delayed recovery [40]. As previously mentioned, changes in endolymph composition may also contribute to the pathophysiology of audiovestibular disorders [41].

Research based on the ultrastructural analysis of the cochlea in an animal model has suggested that the hyperglycemic metabolic status specifically harms the inner ear and this damage does not appear only a consequence of microangiopathy [42]. The authors suggest that this condition may be a metabolically initiated diabetic otopathy, which is characterized by structural and functional alterations of the stria vascularis. Similarly, alterations of the “dark cells” of the vestibular organs, which are mainly involved in potassium exchange, could explain the link between diabetes and vestibular recurrent disease.

The presence of hearing loss among individuals with type 2 diabetes has been reported by a recent meta-analysis, with a range from 40.6% to 71.9% [43]. This finding supports the possibility of a similar involvement of the vestibular organs, although they are not so clinically evident. The validation of an animal model to analyze the vestibular inner ear structure appears more difficult, therefore clinical studies are really important in clarifying the relationship between vestibular disorders and DM.

Aging is another factor that leads to a progressive deterioration of Corti’s organ, with a decrease in hair cell function and reduced efficiency of the transduction system. Vestibular structure and function are known to decline with aging, and age-related vestibular loss is not only related to deficits in posture and balance but also to visuospatial cognitive ability, etc. [44].

Given that chronic diabetic complications are associated with poor glycemic control in several BPPV-focused studies, it was observed that patients with recurrent symptoms had significantly higher HbA1c levels than controls. Moreover, D’Silva et al. [27,28], in a series of studies on patients with BPPV, used HbA1c to characterize metabolic control and disease severity, correlating these parameters with otolithic function assessed through vestibular-evoked myogenic potentials (VEMPs).

The interest in HbA1c extends to auditory disorders as well. Park et al. [35] stratified diabetic patients with SSHL according to HbA1c levels to evaluate the influence of glycemic control on hearing recovery. Overall, these findings suggest that HbA1c represents also a potential prognostic and pathophysiological biomarker in audiovestibular disorders.

By evaluating otherwise healthy vestibular systems using cervical and ocular vestibular-evoked myogenic potentials (cVEMP and oVEMP), other authors demonstrated that patients with type 2 diabetes exhibited reduced response rates, lower response amplitudes, and greater interaural asymmetry compared with healthy controls, suggesting subclinical dysfunction of the otolithic organs. The authors attributed these findings to diabetes-related microangiopathy and hyperglycemia-induced damage to vestibular hair cells [45].

Further confirmation of the close association between DM and vestibular recurrent disease is supported by the use of the nomogram, proposed by several authors as shown in the table. A nomogram is a predictive tool that assigns a corresponding score to each significant risk factor and maps the total score to the probability of recurrence. In this way, clinicians have an available concrete method for individualized risk assessment to develop early intervention strategies in clinical practice.

In light of these considerations, a multidisciplinary clinical approach to patients affected by diabetes and reporting vestibular disorders is recommended especially in older subjects. This applies particularly in relation to the expectations of patients relative to repositioning maneuvers.

The methodological appraisal of the studies included in this review supports the conduct of a systematic review despite the lack of sufficient homogeneity for meta-analysis. Overall, the evidence base was composed of studies with acceptable to high methodological quality, as shown by the Newcastle–Ottawa Scale, the Cochrane RoB 2 tool, and the Joanna Briggs Institute checklist.

Randomized controlled trials were judged to have low or only some concerns for risk of bias, and cross-sectional studies were generally rated as high quality, with only one study showing moderate methodological robustness.

However, this review has some limitations. First, most of the studies analyzed were retrospective and observational, with substantial heterogeneity in the definitions of vertigo, diabetes, outcomes, and follow-up. Moreover, the literature was predominantly focused on BPPV, limiting the generalizability of the findings across the spectrum of vestibular disorders. Furthermore, various studies lacked detailed clinical information regarding diabetes type, disease duration, glycemic control, diabetes-related complications, and, in some cases, the clinical characterization of the vestibular disorder itself. Finally, there is a potential risk of confounding due to age, obesity, dyslipidemia, and other comorbidities.

## 5. Conclusions

The findings of this review suggest an association between DM and BPPV, one of the most frequent vestibular disorders. Most studies identified diabetes as a potential risk factor for disease occurrence, recurrence, or less favorable clinical outcomes. Data regarding other vestibular disorders, including VM and MD, are still limited. Future prospective studies with standardized diagnostic criteria and comprehensive characterization of diabetes status are needed to better clarify the relationship between diabetes and vestibular disorders, beyond BPPV.

## Figures and Tables

**Table 1:** Newcastle–Ottawa Scale (NOS) Assessment.

Study	Total Score (Quality)
Tang et al. [8]	8/9 (High)
Yang et al. [1]	8/9 (High)
Zihihui Du et al. [2]	6/9 (Moderate)
D’Silva [3]	8/9 (High)
Chung et al. [5]	7/9 (High)
Koh et al. [29]	6/9 (Moderate)
Liu et al. [19]	7/9 (High)
Wang et al. [9]	7/9 (High)
Na et al. [21]	7/9 (High)
Luryi et al. [23]	6/9 (Moderate)
Bener et al. [24]	7/9 (High)
Arabian et al. [25]	5/9 (Fair)
Zhou et al. [26]	7/9 (High)
Haremza et al. [32]	6/9 (Moderate)
Hosokawa et al. [37]	7/9 (High)
Zhang et al. [34]	7/9 (High)
Li et al. [35]	9/9 (Very High)
Park et al. [36]	6/9 (Moderate)
Hosokawa et al. [31]	7/9 (High)

**Table 2:** Cochrane Risk of Bias 2 (RoB 2) assessment for randomized controlled trials.

Study	Randomization Process	Deviations from Intended Interventions	Missing Outcome Data	Outcome Measurement	Selective Reporting	Overall Risk of Bias
Shaphe et al. [15]	Low	Some Concerns	Low	Low	Low	Low/Some Concerns
Mehta et al. [33]	Low	Some Concerns	Low	Low	Low	Low

**Table 3:** Joanna Briggs Institute (JBI) critical appraisal checklist for cross-sectional studies.

Study	Inclusion Criteria Clearly Defined	Study Setting and Participants Described	Exposure Measurement	Outcome Measurement	Confounding Factors Identified	Strategies to Address Confounders	Overall Quality
D’Silva et al. [7]	Yes	Yes	Yes (HbA1c/MNSI)	Yes	Yes	Yes	High
Sreenivas et al. [20]	Yes	Yes	Yes	Yes	No	No	Moderate
Bazoni et al. [22]	Yes	Yes	Yes (BMD/Vitamin D)	Yes	Yes	Yes	High
Singh et al. [30]	Yes	Yes		Yes	Yes	Yes	High
D’Silva et al. [27]	Yes	Yes		Yes	Yes	Yes	High
D’Silva et al. [28]	Yes	Yes		Yes	Yes	Yes	High

**Table 4:** Main findings from the 19 studies exploring the link between diabetes and vestibular disorders.

Author and Year	Diabetics	Number of Patient with Vertigo	Cases with Diabetes and Vertigo	Vertigo	Main Findings
Tang B, 2024 [8] China	94	582	94	BPPV, MD	Age, diabetes, and insomnia were independent predictors of BPPV recurrence; an effective predictive nomogram was developed.
Yang T-H., 2024 [1] Taiwan	99,156	150,916	4294	BPPV, MD	Vestibular disorders were significantly associated with diabetes.
Z. Du, 2022 [2] China	31	234	31	VM	Diabetes associated with poorer outcomes.
D’Silva LJ, 2016 [3] USA	699	1518	322	BPPV	Hypertension and type 2 diabetes associated with BPPV.
Liu Y, 2026 [6] China	331	907	331	BPPV	Age, diabetes, and depression predictors of prolonged dizziness; a clinical nomogram successfully validated.
D’Silva LJ, 2017 [7] USA	16	50	16	BPPV	Diabetes did not affect the efficacy of canalith repositioning maneuvers.
Wang P, 2025 [9] China	138	276	138	BPPV	Insomnia, diabetes, and hypertension increased the risk of BPPV recurrence; predictive nomogram facilitated identification of high-risk patients.
Shaphe MA, 2023 [15] South Arabia	30	30	30	BPPV	Epley maneuver and vestibular rehabilitation were effective in diabetic patients.
Huy J., 2024 [16] China	6	6	6	BPPV	Common hub genes identified; immune-mediated inflammatory regulation as a shared pathogenic mechanism.
Shan Liu, 2025 [19] China	(T1DM): 4.721 (T2DM): 82.878	12006	genetic study, no comorbidity data	BPPV	No direct genetic causal relationship between the two types of diabetes and BPPV.
Sreenivas V., 2019 [20] India	8	71	8	BPPV	Age, diabetes, and hypertension increased the risk of BPPV recurrence.
Na S., 2025 [21] South Korea	182	1363	182	BPPV	Secondary BPPV was associated with comorbidities including diabetes.
Bazoni J. 2019 [22] Brazil	25	17	5	BPPV	Association between bone mineral density and BPPV observed only in the diabetic group.
Luryi at al., 2018 [23] USA	134	1105	12.1% of the BPPV sample affected by diabetes	BPPV	Female sex and previous BPPV increased the risk of recurrence; diabetes was not identified as significant risk factors.
Bener A., 2024 [24] Turkey	113	177	21.5% of BPPV had a family history of diabetes	BPPV	A significant correlation between BPPV and DM, significantly higher blood glucose and HbA1c levels.
Arabian R., 2025 [25] USA	32	140	32	BPPV	Patients with BPPV were significantly more likely to have hypothyroidism and DM compared with controls.
Zhou C., 2023 [26] China	30	186	30	BPPV	Age, diabetes, and hypertension were risk factors for residual symptoms; the proposed nomogram demonstrated high predictive accuracy.
D’Silva LJ, 2017 [27] USA	25	24	11	BPPV	Patients with DM and BPPV exhibited greater postural sway.
D’Silva L.J., 2017 [28] USA	33	32	14	BPPV	Diabetes and BPPV independently contributed to vestibular dysfunction.

## Data Availability

No new data were created or analyzed in this study. Data sharing is not applicable to this article.

## Supplementary Materials

The following supporting information can be downloaded at: https://www.mdpi.com/article/10.3390/audiolres16040115/s1. Supplementary File S1: PRISMA checklist.

## Appendix A

Search Strings: ((“Vestibular Migraine”[Mesh] OR “vestibular migraine”[tiab] OR “migraine-associated vertigo”[tiab]) OR (“Benign Paroxysmal Positional Vertigo”[Mesh] OR BPPV[tiab] OR “benign paroxysmal positional vertigo”[tiab]) OR (“Hearing Loss, Sudden”[Mesh] OR SSNHL[tiab] OR “sudden sens OR ineural hearing loss”[tiab]() AND (“Diabetes Mellitus”[Mesh] OR diabetes[tiab] OR “type 1 diabetes”[tiab] OR “type 2 diabetes”[tiab] OR diabetic[tiab)) NOT (“Presbycusis”[Mesh] OR presbycusis[tiab]) NOT (animals[Mesh] NOT humans[Mesh]) NOT (mice[tiab] OR mouse[tiab] OR rat[tiab] OR rats[tiab] OR “in vitro”[tiab] OR “cell line”[tiab]) AND (“2016/01/01”[Date—Publication]: “3000”[Date—Publication))((“VestibularMigraine”[Mesh] OR “vestibular migraine”[tiab] OR “migraine-associated vertigo”[tiab] OR “migraine related vertigo”[tiab]) OR (“Benign Paroxysmal Positional Vertigo”[Mesh] OR BPPV[tiab] OR “benign paroxysmal positional vertigo”[tiab] OR “positional vertigo”[tiab]) OR (“Hearing Loss, Sudden”[Mesh] OR SSNHL[tiab] OR “sudden sens OR ineural hearing loss”[tiab] OR “sudden hearing loss”[tiab])) AND (“Diabetes Mellitus”[Mesh] OR “Diabetes Mellitus, Type 2”[Mesh] OR “Diabetes Mellitus, Type 1”[Mesh] OR diabetes[tiab] OR diabetic[tiab]) NOT (“Presbycusis”[Mesh] OR presbycusis[tiab] OR “age-related hearing loss”[tiab]) NOT (animals[Mesh] NOT humans[Mesh]) NOT (“in vitro”[tiab] OR mice[tiab] OR mouse[tiab] OR rat[tiab] OR rats[tiab]) AND (“2016/01/01”[Date—Publication]: “3000”[Date—Publication]).
